# Supplementary material for: Cryopreservation of Sperm from an Endangered Snake with Tests of Post-Thaw Incubation in Caffeine
Source: Animals (Basel). 2022 Jul 17;12(14):1824. doi: 10.3390/ani12141824 (PMC9311608; doi:10.3390/ani12141824)
Supplement: Supplementary file 1 [file animals-12-01824-s001.zip › animals-1772958-supplementary.pdf]

Supplementary Tables and Figures

Table S1. Summary table of mean values (± s.d.) of motility and membrane viability measures of thawed semen samples without dilution ("raw") across the control CPA group held constant in each experimental CPA group (A, B, C, D). Below mean values are the results of Kruska-Wallis test of differences for each semen metric included in the table. (M = % motile sperm, MF = % sperm moving forward, % motile MF = proportion of motile sperm moving forward, FPM = forward progressive motility LD = membrane viability)

Control CPA	N	M	MF	% motile MF	FPM	Total Motility	Recovered LD
A	4	9.8 ± 4.4	0	0	0	9.8 ± 4.4	26.9 ± 8.3
B	4	12.5 ± 6.4	0	0	0	12.5 ± 6.4	30.8 ± 24.5
C	5	13.4 ± 6.6	0	0	0	13.4 ± 6.6	35.9 ± 1.5
D	4	14.1 ± 10.9	0	0	0	14.1 ± 10.9	19.5 ± 8.0
	H value	1.39	NA	NA	NA	1.39	5.23
	d.f.	3	3	3	3	3	3
	P value	0.708	NA	NA	NA	0.708	0.156
